# Functionalized magnetic hydrogel encapsulation of human dental follicle stem cells under a static magnetic field enhances multi-site bone regeneration

**DOI:** 10.1093/rb/rbag023

**Published:** 2026-03-07

**Authors:** Peishen Deng, Manhong Zheng, Bing Du, Changyu Liu, Renyi Cheng, Chaofeng Liu, Fang Wang, Hangyu Dong, Yan Shan, Yanhua Xu

**Affiliations:** Yunnan Key Laboratory of Stomatology & Department of Orthodontics, The Affiliated Stomatology Hospital, Kunming Medical University, Kunming 650106, China; Department of Orthodontics, Stomatology Hospital of Yunnan Province, Kunming 650106, China; Department of Stomatology, The Second People’s Hospital of Foshan,Affiliated Foshan Hospital of Guangdong Pharmaceutical University, Foshan 528000, Guangdong Province, China; Yunnan Key Laboratory of Stomatology & Department of Periodontics, The Affiliated Stomatology Hospital, Kunming Medical University, Kunming 650106, China; Department of Periodontics, Stomatology Hospital of Yunnan Province, Kunming 650106, China; Department of Stomatology, The Second People’s Hospital of Foshan,Affiliated Foshan Hospital of Guangdong Pharmaceutical University, Foshan 528000, Guangdong Province, China; Yunnan Key Laboratory of Stomatology & Department of Orthodontics, The Affiliated Stomatology Hospital, Kunming Medical University, Kunming 650106, China; Department of Orthodontics, Stomatology Hospital of Yunnan Province, Kunming 650106, China; Yunnan Key Laboratory of Stomatology & Department of Orthodontics, The Affiliated Stomatology Hospital, Kunming Medical University, Kunming 650106, China; Department of Orthodontics, Stomatology Hospital of Yunnan Province, Kunming 650106, China; Yunnan Key Laboratory of Stomatology & Department of Orthodontics, The Affiliated Stomatology Hospital, Kunming Medical University, Kunming 650106, China; Department of Orthodontics, Stomatology Hospital of Yunnan Province, Kunming 650106, China; Yunnan Key Laboratory of Stomatology & Department of Orthodontics, The Affiliated Stomatology Hospital, Kunming Medical University, Kunming 650106, China; Department of Orthodontics, Stomatology Hospital of Yunnan Province, Kunming 650106, China; Yunnan Key Laboratory of Stomatology & Department of Orthodontics, The Affiliated Stomatology Hospital, Kunming Medical University, Kunming 650106, China; Department of Orthodontics, Stomatology Hospital of Yunnan Province, Kunming 650106, China; Yunnan Key Laboratory of Stomatology & Department of Orthodontics, The Affiliated Stomatology Hospital, Kunming Medical University, Kunming 650106, China; Department of Orthodontics, Stomatology Hospital of Yunnan Province, Kunming 650106, China; Yunnan Key Laboratory of Stomatology & Department of Orthodontics, The Affiliated Stomatology Hospital, Kunming Medical University, Kunming 650106, China; Department of Orthodontics, Stomatology Hospital of Yunnan Province, Kunming 650106, China

**Keywords:** human dental follicle stem cells, magnetic nanoparticles, gelatin methacryloyl hydrogel, static magnetic field, multi-site bone regeneration

## Abstract

Repairing large-scale craniomaxillofacial bone defects is hindered by a limited availability of stem-cell sources and a low osteogenic efficiency. To address these challenges, Fe_3_O_4_ nanoparticles were modified with methacrylic anhydride (MAA), which helped to introduce photopolymerizable methacryloyl groups, resulting in MAA–Fe_3_O_4_ nanoparticles that exhibit excellent magnetic properties and colloidal stability. These nanoparticles were incorporated into gelatin methacryloyl (GelMA) and covalently crosslinked to form an injectable, photocurable GelMA–Fe_3_O_4_ magnetic composite hydrogel. This hydrogel provided a three-dimensional culture microenvironment for human dental follicle stem cells (hDFSCs), and upon encapsulation, osteogenesis was significantly enhanced under a 100 mT static magnetic field (SMF). *In vitro*, GelMA–Fe_3_O_4_ hydrogels demonstrated increased porosity and improved mechanical properties, thereby significantly promoting hDFSCs proliferation, adhesion and spreading. Additionally, under SMF exposure, the expression of osteogenesis-related genes and proteins, including alkaline phosphatase (ALP), Runx2, Col-I and OPN, was significantly upregulated. In a rat calvarial defect model, bone mineralization centers with multi-site distribution were observed in the GelMA–Fe_3_O_4_ + SMF group as early as 4 weeks postoperatively, leading to high-quality defect repair. The limitations of traditional ‘peripheral-to-center’ unidirectional repair were overcome by this model of synchronous multi-site osteogenesis, maximizing bone regeneration with a minimal number of stem cells and providing an efficient, controllable tissue-engineering strategy for the clinical treatment of craniomaxillofacial bone defects.

## Graphical abstract

**Graphical Abstract:**
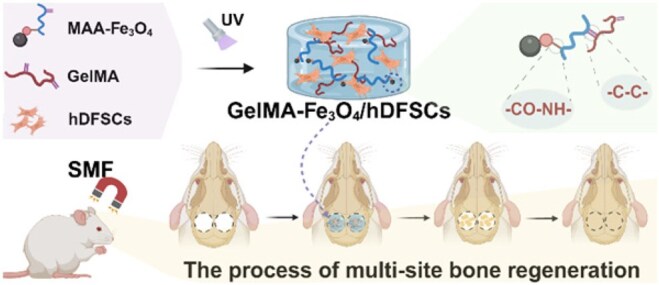

## Introduction

Mesenchymal stem cells (MSCs) hold substantial therapeutic promise for bone tissue engineering due to their multipotent differentiation capacity and self-renewal [1]. Compared to bone marrow- and adipose tissue-derived MSCs, dental-derived MSCs—particularly human dental follicle stem cells (hDFSCs)—have garnered increasing attention because of their unique advantages [2]. hDFSCs originate from follicular tissue during the early stages of tooth development and share developmental similarities with craniofacial bones. They exhibit bidirectional differentiation toward osteogenesis and odontogenesis, along with enhanced *in vitro* proliferation, delayed senescence and robust immunomodulatory activity [3, 4]. Importantly, hDFSCs can be obtained non-invasively during routine clinical procedures (e.g. wisdom tooth extraction) with minimal morbidity, providing a readily accessible cell source for clinical applications [5]. However, hDFSC-based bone repair strategies still face several challenges: limited intraconstruct cell distribution within three-dimensional (3D) scaffolds; insufficient osteogenic efficacy with low-dose transplantation; an increased risk of immune responses with high-dose transplantation [6, 7]; and the traditional “peripheral-to-center” unidirectional osteogenic paradigm, which results in inefficient repair [8]. Collectively, these constraints lead to suboptimal repair outcomes.

While conventional bone repair materials provide essential structural support, they are limited in their ability to direct lineage-specific differentiation and modulate the osteogenic microenvironment. In recent years, magnetic nanoparticles, particularly magnetite (Fe_3_O_4_), have garnered significant interest in the field of bone tissue engineering due to their favorable biocompatibility, biodegradability, and magnetoresponsive properties [9]. Under a static magnetic field (SMF), Fe_3_O_4_ nanoparticles facilitate MSCs’ osteogenesis and angiogenesis through magneto-mechanical transduction [10, 11]. However, their poor surface hydrophilicity and tendency to agglomerate result in heterogeneous dispersion of Fe_3_O_4_ within polymeric matrices and weak interfacial bonding, thereby limiting their efficacy in composite applications [12].

Hydrogels, which mimic the native extracellular matrix, provide an effective platform to address these issues [13, 14]. Gelatin methacryloyl (GelMA) hydrogels are widely used scaffolds in bone tissue engineering due to their biocompatibility, tunable mechanical properties and photocrosslinkable chemistry [15]. However, the crosslinking density of neat GelMA is often inadequate, resulting in mechanical strength that does not meet the requirements for large-scale bone defect repair [16]. Additionally, nanoparticles tend to distribute heterogeneously and form weak interfacial interactions with the polymer network during hydrogel formation [17]. To overcome these limitations, Fe_3_O_4_ nanoparticles were surface-functionalized with methacrylic anhydride (MAA) to introduce methacryloyl groups capable of participating in free-radical photopolymerization, thus yielding MAA–Fe_3_O_4_ nano-crosslinkers. This strategy enhances the hydrophilicity and dispersibility of the nanoparticles and facilitates synergistic photocrosslinking with the GelMA matrix, substantially increasing network crosslinking density, improving mechanical performance and augmenting the magneto-responsive bioactivity of the composite hydrogel.

SMF has garnered increasing attention as a non-invasive physical stimulus in the field of bone tissue engineering [18]. SMF promotes the osteogenic differentiation of MSCs by modulating intracellular calcium levels and regulating osteogenesis-related signaling pathways, notably those mediated by Smad4 [19, 20]. When combined with magnetic nanoparticles, SMF produces pronounced synergistic effects; it drives Fe_3_O_4_ nanoparticles to deliver microscale mechanical cues that activate osteogenic programs through mechanotransduction, while also inducing their directional alignment to form ordered architectures that create an anisotropic microenvironment conducive to cell growth [21, 22]. This triad of SMF, nanoparticles and cells presents a promising strategy for enhancing the efficiency of bone repair.

**Figure 1:**
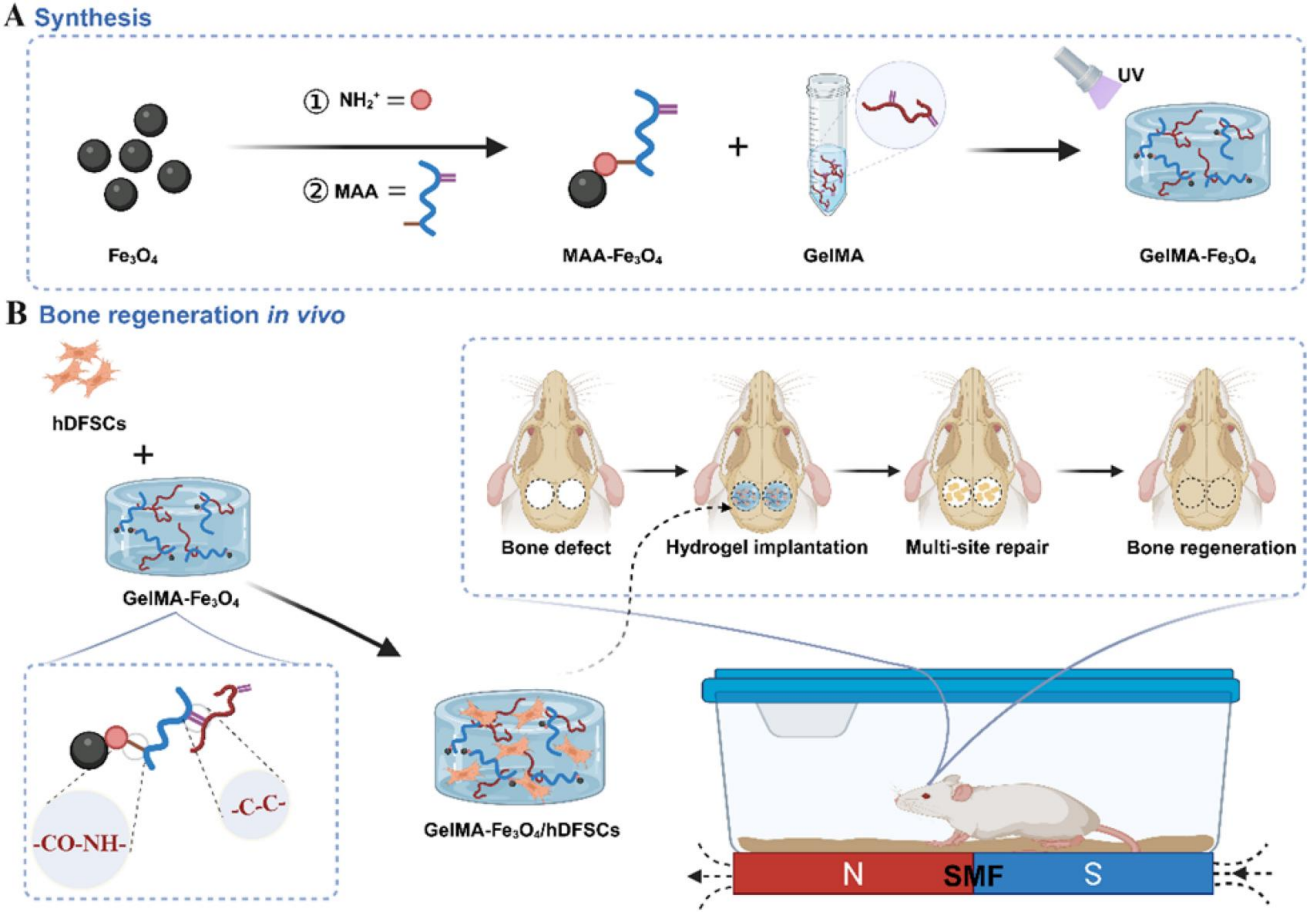
Schematic illustration of the construction and application of cell‑laden GelMA–Fe_3_O_4_ 3D hydrogel scaffolds. (**A**) The scaffold consists of a GelMA hydrogel network and methacrylic acid–modified Fe_3_O_4_ nanoparticles. (**B**) The scaffold encapsulates hDFSCs and promotes the repair of rat calvarial defects under an external magnetic field.

Building on this background, the present study was designed to address the application bottlenecks of hDFSCs in bone repair, as well as the functional limitations of conventional hydrogel scaffolds. To this end, we developed an injectable, photo-crosslinkable GelMA–Fe_3_O_4_ magnetically functionalized hydrogel system. MAA-functionalized Fe_3_O_4_ nanoparticles were covalently crosslinked with GelMA to construct a 3D scaffold that exhibits enhanced magnetic responsiveness and mechanical properties. Subsequently, hDFSCs were loaded into this scaffold to form a cell-material composite. The effects of this system on hDFSC proliferation, migration and osteogenic differentiation were evaluated *in vitro*, with particular attention to the synergistic interactions between an SMF and the magnetic hydrogel. Furthermore, the *in vivo* bone-repair efficacy was validated in a rat calvarial defect model (Figure 1). It is anticipated that this magnetically functionalized hydrogel will facilitate the formation of multi-site osteogenic centers under an SMF, thereby overcoming the limitations of traditional unidirectional osteogenic models. This approach is expected to enhance the spatial utilization efficiency and osteogenic efficacy of hDFSCs, providing a controllable and efficient tissue-engineering strategy for the clinical treatment of craniomaxillofacial bone defects.

## Materials and methods

### Sample preparation

#### Functionalization and modification methods of nanoparticles

1. Amination modification: Fe_3_O_4_ nanoparticles (20 mg; Innochem) were dispersed in 20 mL of deionized water (Biosharp) and sonicated for 10 minutes to achieve a homogeneous suspension. Ethylenediamine (EDA, 0.2 mL; Aladdin) was then added while stirring at 500 rpm, and the pH was adjusted to 9–10 through the dropwise addition of ammonia solution (25–28%, 0.2–1 mL; Aladdin). The reaction was conducted at 80°C with stirring at 300 rpm for 6 hours (stirring speed was adjusted to optimize high-temperature reaction conditions). The nanoparticles were collected via centrifugation (10 000 × g for 10 minutes), washed three times with deionized water, and subsequently lyophilized at −80°C (FreeZone 2.5 L; LabConco) for storage.
2. MAA modification: MAA (Sigma-Aldrich) was dissolved in 50 mM MES buffer (Aladdin) to achieve a final concentration of 15 mM while kept on ice. EDC·HCl and NHS (both at 15 mM; Aladdin) were subsequently added, and the mixture was stirred in the dark for 20 minutes. Aminated Fe_3_O_4_ (20 mg) was dispersed in 50 mM MES buffer (pH 5.5; total volume of 20 mL) and then combined with the activated solution. The pH was gradually adjusted to 7.2–7.4 using 50 mM HEPES buffer (Macklin). The reaction was allowed to proceed at room temperature (RT) with gentle stirring in the dark for 6 hours. The reaction was terminated by adding Tris (1 M, pH 8.0) to a final concentration of 20 mM. The final product was collected via centrifugation (10 000 × g for 10 minutes), washed three times with deionized water, and freeze-dried to obtain MAA–Fe_3_O_4_ nanoparticles.

#### Synthesis of GelMA hydrogel

GelMA (1.0 g, EFL) was dissolved in sterile phosphate-buffered saline (PBS, 10 mL; Biosharp). Lithium phenyl-2,4,6-trimethylbenzoylphosphinate (LAP; 0.25% w/v, EFL) was incorporated as a photoinitiator. The mixture was then magnetically stirred in a 65°C water bath, protected from light, for 15 minutes until a clear solution was achieved, resulting in a 100 mg/mL GelMA precursor solution.

#### Preparation of composite hydrogels

At the desired final mass concentrations (mg/mL), MAA–Fe_3_O_4_ nanoparticles (at different concentrations) were uniformly dispersed in the GelMA precursor solution. Following gentle stirring at 65°C in the dark for 20 minutes, the mixture was injected into standardized silicone molds to ensure all hydrogel composites had a consistent cylindrical shape and dimensions. The mixture was photocrosslinked under 405 nm visible (violet) light (10 mW/cm^2^) for 1 minute to produce GelMA–Fe_3_O_4_ composite hydrogels (10-mm diameter, 10-mm-thick cylinders for subsequent physicochemical characterization). As a control, an equal amount of unmodified Fe_3_O_4_ nanoparticles was mixed with GelMA in the same ratio and processed under identical conditions to prepare GelMA/Fe_3_O_4_ control hydrogels. All samples (GelMA, GelMA/Fe_3_O_4_, GelMA–Fe_3_O_4_) were lyophilized after being pre-frozen at −80°C for 48 hours and then sterilized by gamma irradiation for later use.

### Material characteristics

#### Nanoparticle characterization

The morphology, crystalline phase and chemical composition of Fe_3_O_4_ and MAA–Fe_3_O_4_ nanoparticles were characterized using transmission electron microscopy (TEM; Tecnai G2 F20, FEI, USA), X-ray diffraction (XRD; SmartLab, Rigaku, Japan), and Fourier-transform infrared (FTIR) spectroscopy (VERTEX 70, Bruker, Germany). The zeta potential was assessed through electrophoretic light scattering with a Zetasizer Nano ZS (Malvern Panalytical, UK), while the magnetic properties were evaluated using a vibrating sample magnetometer (VSM; 7404, Lake Shore Cryotronics, USA). All experimental data were analyzed using Origin (OriginLab).

#### Characterization of composite hydrogels

The microstructure and elemental distribution of GelMA/Fe_3_O_4_ and GelMA–Fe_3_O_4_ composite hydrogels were analyzed using scanning electron microscopy (SEM) and energy-dispersive X-ray spectroscopy (EDS; X-Max, Oxford Instruments, UK). Porosity was calculated from SEM images with ImageJ (NIH). The crystalline structure and chemical composition were characterized through XRD and FTIR spectroscopy, respectively, while the magnetic properties were assessed using a VSM. *In vitro* degradation was evaluated by monitoring changes in dry weight in PBS at 37°C on days 7, 14, 21, 28, 35, 42 and 49, with the degradation rate calculated using the formula DR = (W_0_ − Wₜ)/W_0_ × 100%, and the morphological changes of hydrogels were observed by SEM at days 0, 15 and 60. The swelling behavior was determined after 24 hours of equilibration, with the swelling ratio calculated as SR = (Ww − Wd)/Ww × 100%. The mechanical properties, including compressive strength and modulus, were tested using a universal testing machine (AGS-X, Shimadzu, Japan).

### *In vitro* experiment

#### Isolation and identification of hDFSCs

This study received approval from the Ethics Committee of the Affiliated Stomatological Hospital of Kunming Medical University (KYKQ2024MEC0070; February 28, 2024). Written informed consent was obtained from orthodontic patients aged 18–25 years prior to the collection of third molar dental follicle tissues. The tissue blocks were sequentially digested with collagenase type I (Sigma-Aldrich, USA) followed by 0.25% trypsin (Gibco, USA), and cells were subsequently collected by centrifugation. The cells were cultured in α-MEM (Gibco) supplemented with 20% fetal bovine serum (FBS; Gibco) at 37°C in a humidified atmosphere containing 5% CO_2_. Cells at passages 3–5 were utilized for subsequent experiments. Flow cytometry was employed to identify the MSC phenotype, characterized by CD44+/CD105+ and CD34−/CD45−. The self-renewal capacity was assessed using a colony-forming unit assay, with colonies stained using 0.1% crystal violet (Solarbio). Osteogenic and adipogenic differentiation potentials were confirmed through Alizarin Red S and Oil Red O staining, respectively (both from Beyotime).

#### SMF device

SMF was generated using neodymium–iron–boron (NdFeB) permanent magnets with a diameter of 10 mm (Ningbo Tianhe Magnetism Industry, China). After calibrating the magnetic flux density (B) with a gaussmeter (Kanetec, Japan), an adjustable SMF ranging from 50 to 400 mT at the sample position was achieved by stacking 2-mm-thick, 10-mm-diameter magnetic discs and/or adjusting the distance between the magnet and the sample. All magnetic field exposures were conducted in a 37°C, 5% CO_2_ incubator to ensure a consistent temperature and gas composition.

#### Cytotoxicity and proliferation assays

The cytotoxicity and proliferative effects of the materials on hDFSCs were systematically evaluated using the Cell Counting Kit-8 (CCK-8, Beyotime). The experiments were categorized into two-dimensional (2D) and 3D culture systems.

1. 2D culture system: cells were seeded at a density of 3 × 10^4^ cells/well, and MAA–Fe_3_O_4_ was added at concentrations ranging from 0 to 200 μg/mL. The culture was maintained for 1, 3 and 7 days; 10 μL of CCK-8 was added to each well, followed by incubation at 37°C for 1 hour. The absorbance was measured at a wavelength of 450 nm using a Thermo Multiskan FC.
2. 3D culture system: cells (5 × 10^4^ cells/mL) were combined with a GelMA precursor solution containing 0–800 μg/mL MAA–Fe_3_O_4_, photo-cured at 405 nm visible (violet) light for 20 seconds, to prepare 3D culture hydrogels (cylindrical, 6-mm diameter, 2-mm thickness) and cultured in three dimensions. Processing was conducted on days 1, 3 and 7 as described above for the CCK-8 assay.
3. Magnetic field intervention experiment: in the 400 μg/mL MAA–Fe_3_O_4_ hydrogel, SMF of 0, 50, 100, 200 and 400 mT were applied, respectively. The control groups included GelMA/Fe_3_O_4_, GelMA–Fe_3_O_4_ and GelMA–Fe_3_O_4_ with 100 mT SMF. The CCK-8 assay was employed to assess proliferation differences. Each group consisted of at least three replicate wells, and the data were expressed as mean ± standard deviation (SD).

#### Cell viability and morphology

1. Live/dead staining: the viability of hDFSCs in the hydrogel was evaluated using the Live/Dead Cell Staining Kit (Beyotime) on days 1, 3 and 7 of co-culture. After washing the samples with PBS, a staining solution containing calcein AM (2 μM) and propidium iodide (4 μM) was applied, followed by incubation at 37°C in the dark for 20 minutes. Live cells, exhibiting green fluorescence, and dead cells, exhibiting red fluorescence, were observed using confocal laser scanning microscopy (CLSM) (Olympus FV3000).
2. Cytoskeleton staining: Phalloidin staining was conducted on days 1 and 7. Following fixation with 4% paraformaldehyde (PFA), the samples were permeabilized using 0.1% Triton X-100 and stained with phalloidin (1:100) in the dark for 1 hour. Subsequently, the samples were counterstained with DAPI to visualize the nuclei. The morphology of the cytoskeleton was analyzed using CLSM.

#### Cell adhesion and nanoparticle uptake

hDFSCs at a density of 5 × 10^4^ cells per well were seeded in GelMA–Fe_3_O_4_ containing 400 μg/mL MAA–Fe_3_O_4_ and cultured in a 3D environment for durations of 1 and 7 days. Subsequently, the cells were fixed using 2.5% (w/v) glutaraldehyde at 4°C for 2 hours, followed by dehydration through a gradient of ethanol. The cell adhesion and micro-morphology were assessed using SEM. Additionally, samples co-cultured for 3 days were collected, and 200 μL of GelMA lysis buffer (EFL) was added per well. The samples were then subjected to centrifugation at 1000 × g to harvest the cells. After fixation with glutaraldehyde and embedding in resin, the intracellular distribution of nanoparticles was analyzed using TEM.

#### Alkaline phosphatase and alizarin red staining

The hDFSCs–hydrogel composite was cultured in a 3D environment using osteogenic induction medium (Beyotime). The GelMA–Fe_3_O_4_ + 100 mT SMF group underwent continuous magnetic field stimulation, with the medium being replaced every 3 days.

ALP staining: on days 7 and 14, samples were fixed with 4% PFA for 30 minutes, developed according to the protocol provided in the Beyotime ALP kit, and subsequently imaged using an inverted microscope (Nikon, Japan).

ALP activity quantification: on days 7 and 14, cells were lysed with 0.1% Triton X-100 for 2 hours, and ALP enzyme activity was determined using the p-nitrophenyl phosphate substrate method at 405-nm wavelength and normalized to total protein content using a BCA protein quantification kit.

Alizarin Red S staining: on days 14 and 21, samples were stained with 0.1% Alizarin Red S (Beyotime) for 20 minutes, followed by washing with PBS, and the calcified nodules were observed using a Nikon microscope.

#### Immunofluorescence staining

hDFSCs were seeded into different groups of hydrogels to prepare hDFSCs-hydrogel composites and placed in six-well plates with sterilized coverslips for 3D culture, with the GelMA–Fe_3_O_4_ + SMF group receiving continuous 100 mT magnetic field stimulation. When the cells reached approximately 50% confluence, they were fixed with 4% PFA at RT for 15 minutes, permeabilized with 0.5% Triton X-100 for 10 minutes, and subsequently blocked with 5% bovine serum albumin for 30 minutes. Following these steps, the cells were incubated with a primary antibody against collagen type I (COL-I) (Abclonal A1352, 1:200) at 4°C overnight. After washing with PBS, the cells were incubated with a Coralite 488-labeled goat anti-rabbit secondary antibody (1:200; Beyotime) at RT in the dark for 1 hour, followed by DAPI nuclear staining for 5 minutes. Finally, images were acquired using a Nikon A1-SI confocal microscope equipped with NIS-Elements software.

#### Quantitative real-time polymerase chain reaction analysis

The 3D co-cultures were incubated in osteogenic induction medium for 7 and 14 days (*n* = 3). Total RNA was extracted using Trizol (Takara), and complementary DNA (cDNA) was synthesized via reverse transcription (Takara). The expression levels of ALP, COL-I, runt-related transcription factor 2 (RUNX2) and osteopontin (OPN) genes were quantified using the primers listed in Table 1 and the SYBR Green qPCR Kit (Takara) on the Applied Biosystems 7300 system.

**Table 1:** Primer sequences.

Genes	Sequences	
GAPDH	F: CTTTGGTATCGTGGAAGGACTC	R: GTAGAGGCAGGGATGATGTTCT
COL-1	F: AACATGGAGACTGGTGAGACCT	R: CGCCATACTCGAACTGGAATC
ALP	F: TAAGGACATCGCCTACCAGCTC	R: TCTTCCAGGTGTCAACGAGGT
RUNX2	F: CTTTACTTACACCCCGCCAGTC	R: AGAGATATGGAGTGCTGGTC
OPN	F: CTCCATTGACTCGAACGACTC	R: CAGGTCTGCGAAACTTCTTAGA

#### Western blot analysis

After 14 days of osteogenic induction, proteins were extracted using ice-cold RIPA lysis buffer (Solarbio) and quantified via the BCA assay. A total of 30 μg of protein was separated by 10% SDS-PAGE (Lijie, Shanghai), transferred to a polyvinylidene fluoride (PVDF) membrane, and blocked with 5% skim milk for 1 hour. Antibody incubation was conducted as follows: ALP (Proteintech, 1:1000), COL-I (Proteintech, 1:1000), OPN (Proteintech, 1:1000), and GAPDH (Proteintech, 1:5000) were incubated at 4°C overnight; the horseradish peroxidase (HRP)-conjugated goat anti-rabbit secondary antibody (1:5000, Proteintech) was incubated at RT for 1 hour. Following ECL development, the protein band intensity was analyzed semi-quantitatively using ImageJ.

### *In vivo* experiment

#### Animal models and surgery

This study was approved by the Animal Experiment Ethics Committee of Kunming Medical University (kmmu20241921; November 12, 2024). Forty-eight SD rats (200–250 g) were randomly divided into six groups (*n* = 8): blank control, GelMA, GelMA + hDFSCs, GelMA/Fe_3_O_4_ + hDFSCs, GelMA–Fe_3_O_4_ + hDFSCs and GelMA–Fe_3_O_4_ + hDFSCs + SMF. The SD rats were anesthetized using 1% sodium pentobarbital (i.p.), and the surgical area was routinely disinfected with iodophor. An incision was made along the sagittal suture of the skull to expose the cranial top, and a critical-sized defect was created using a 5-mm trephine. With the exception of the blank group, each group was implanted with a 3D cell-hydrogel complex (5 mm diameter, 2 mm thickness) (cells at a concentration of 5 × 10^4^ cells/mL were mixed with a GelMA precursor solution containing 800 μg/mL MAA–Fe_3_O_4_, Fe_3_O_4_, or without nanoparticles, photocured at 405 nm for 20 seconds, and placed in an incubator at 37°C, 5% CO_2_ for 3D co-culture for 7 days, with medium replacement every 3 days). From day 5 post-operation, penicillin (10 000 U/day) was administered intramuscularly to prevent infection; the SMF group was continuously exposed to an SMF by placing a magnet plate (100 mT), calibrated with a gaussmeter, at the bottom of the cage.

#### Micro-CT evaluation

At the 4th and 8th weeks post-operation, euthanasia was performed to harvest cranial specimens. These specimens were subsequently scanned using micro-CT (Skyscan 1276, Bruker, Belgium) at a voltage of 50 kV, a current of 200 μA, and a resolution of 10 μm. A cylindrical region of interest with a diameter of 5 mm was established, followed by 3D reconstruction and quantification of bone volume fraction (BV/TV), bone mineral density (BMD), trabecular thickness (Tb.Th), and bone surface (BS).

#### Histology and immunofluorescence

The specimens, after undergoing Micro-CT scanning, were fixed in 4% PFA, decalcified using a 10% EDTA solution, and subsequently embedded in paraffin for routine sectioning. Hematoxylin and eosin (H&E) staining, along with Masson’s trichrome staining (Solarbio), was performed to evaluate bone repair and the condition of surrounding soft tissues. At 4 and 8 weeks post-operation, heart, liver, spleen, lung and kidney tissues were collected and subjected to H&E staining to assess systemic biocompatibility. Sections from the bone defect area were immunostained with OPN, osteocalcin (OCN) and COL-I antibodies (ServiceBio), and images were captured using a fluorescence microscope (Nikon). The expression levels of target proteins were semi-quantified utilizing ImageJ software.

### Statistical analysis

All experiments were conducted with a minimum of three independent replicates, and the data are presented as mean ± SD. Differences between groups were analyzed using one-way ANOVA, with significance levels established at *P < 0.05, **P < 0.01, ***P < 0.001 and ****P < 0.0001.

## Experimental results

### Characterization of methacrylic acid-modified Fe_3_O_4_

**Figure 2:**
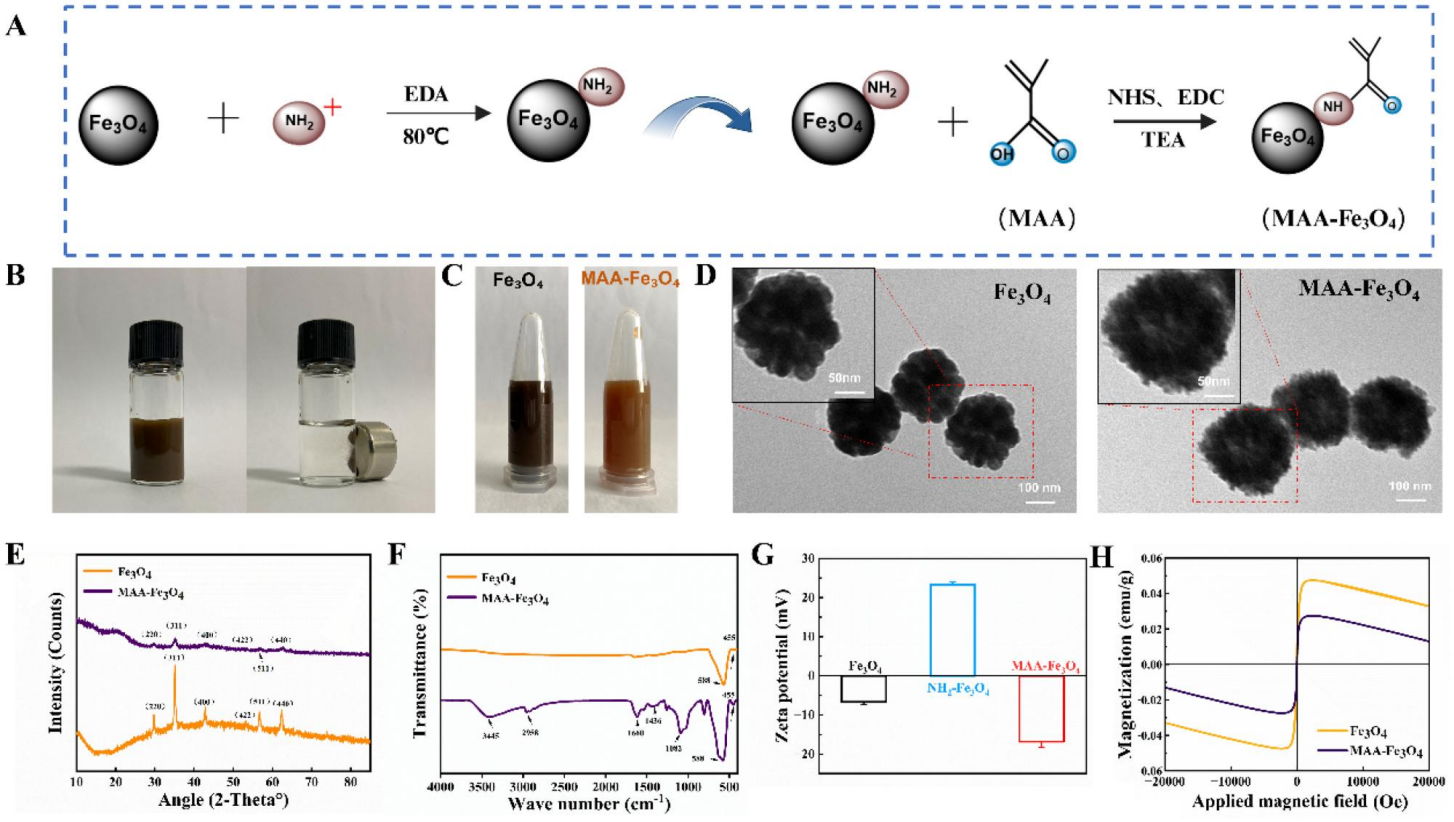
Characterization of Fe_3_O_4_ and MAA–Fe_3_O_4_ nanoparticles. (**A**) Schematic illustration of the preparation of MAA–Fe_3_O_4_ nanoparticles. (**B**) Magnetic response of MAA–Fe_3_O_4_ nanoparticles in anhydrous ethanol. (**C**) Dispersion states of Fe_3_O_4_ and MAA–Fe_3_O_4_ nanoparticles at the same concentration in anhydrous ethanol. (**D**) TEM images of Fe_3_O_4_ and MAA–Fe_3_O_4_ nanoparticles (scale bar = 100 nm. Inset: 50 nm). (**E**) XRD diffraction patterns. (**F**) FTIR spectra of Fe_3_O_4_ and MAA–Fe_3_O_4_ nanoparticles. (**G**) Zeta potential distributions of Fe_3_O_4_, NH_2_-Fe_3_O_4_, and MAA–Fe_3_O_4_ nanoparticles. (**H**) Magnetization curves of Fe_3_O_4_ and MAA–Fe_3_O_4_ nanoparticles.

The NH_2_-Fe_3_O_4_ intermediate was prepared by modifying the surface of Fe_3_O_4_ nanoparticles with amino groups. Subsequently, MAA–Fe_3_O_4_ nanoparticles were synthesized via an amidation reaction between NH_2_-Fe_3_O_4_ and MAA (Figure 2A). The MAA–Fe_3_O_4_ nanoparticles demonstrated superparamagnetic properties, were stably dispersed in anhydrous ethanol, and exhibited a rapid response to an external magnetic field (Figure 2B). At the same mass concentration, the Fe_3_O_4_ suspension changed from a deep black to a translucent brown after MAA modification (Figure 2C). The TEM revealed that the Fe_3_O_4_ nanoparticles were uniform spherical particles with an average diameter of 100 nm. Following MAA modification, the morphology remained spherical; however, a polymer coating layer formed on the surface, and the particle size slightly increased, indicating successful grafting of MAA onto the surface of the Fe_3_O_4_ nanoparticles (Figure 2D). The XRD pattern indicated that the Fe_3_O_4_ nanoparticles exhibited six characteristic peaks of inverse spinel both before and after modification, suggesting that MAA functionalization did not alter the crystal phase of Fe_3_O_4_ (Figure 2E). The FTIR results demonstrated that while the Fe–O absorption peak (588 cm^−1^) was retained, new stretching vibration peaks for C=O (1660 cm^−1^) and –COO^−^ (1436, 1082 cm^−1^) appeared in MAA–Fe_3_O_4_, confirming the successful grafting of MAA onto the Fe_3_O_4_ nanoparticles (Figure 2F). Zeta potential measurements (Figure 2G) showed that the surface charge changed from –6.71 mV for Fe_3_O_4_ to +23.27 mV for NH_2_-Fe_3_O_4_, and subsequently to –16.83 mV for MAA–Fe_3_O_4_, reflecting the changes in potential during the stepwise surface modification from amination to MAA grafting. The VSM test (Figure 2H) demonstrated that, although the organic coating layer slightly reduced the saturation magnetization, the MAA–Fe_3_O_4_ nanoparticles continued to maintain their superparamagnetic characteristics.

### Preparation and characterization of functionalized GelMA–Fe_3_O_4_ composite hydrogel

**Figure 3:**
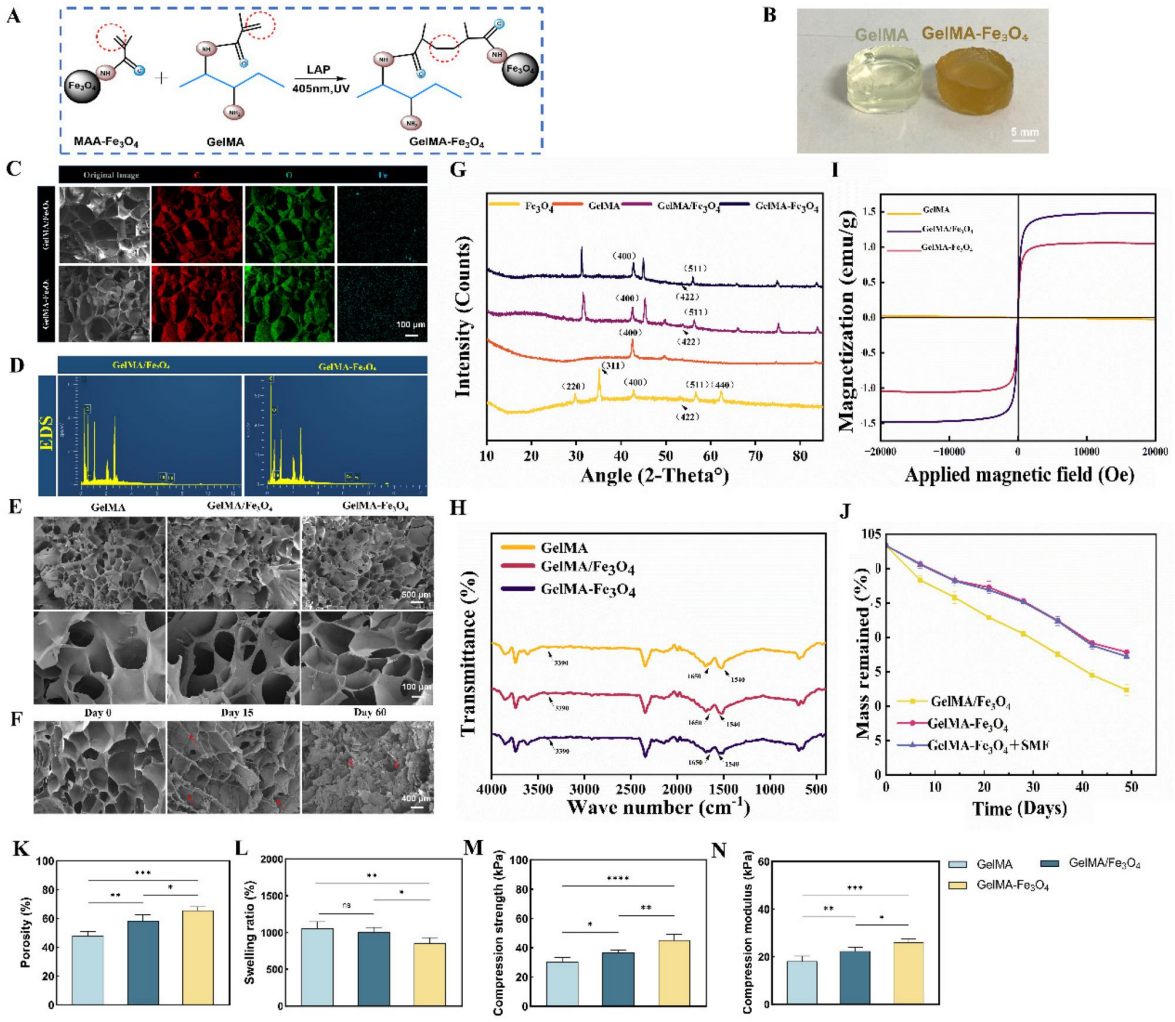
Characterization of different hydrogels. (**A**) Schematic diagram of the preparation process of GelMA–Fe_3_O_4_ hydrogel. (**B**) Appearance of GelMA and GelMA–Fe_3_O_4_ hydrogels after photocrosslinking and curing (scale bar = 5 mm). (**C**) EDS elemental distribution maps of GelMA/Fe_3_O_4_ and GelMA–Fe_3_O_4_ hydrogels (scale bar = 100 μm). (**D**) EDS spectra of the two composite hydrogels mentioned above. (**E**) SEM images of GelMA, GelMA/Fe_3_O_4_ and GelMA–Fe_3_O_4_ hydrogels. Upper row scale bar = 500 μm. Lower row scale bar = 100 μm. (**F**) SEM images of the internal structure of GelMA–Fe_3_O_4_ hydrogel at degradation days 0, 15 and 60 (scale bar = 400 μm). (**G**) XRD patterns of Fe_3_O_4_, GelMA, GelMA/Fe_3_O_4_ and GelMA–Fe_3_O_4_ hydrogels. (**H**) FTIR spectra of the three hydrogels. (**I**) Magnetization curves of the three hydrogels. (**J**) *In vitro* degradation rates of GelMA/Fe_3_O_4_, GelMA–Fe_3_O_4_ and GelMA–Fe_3_O_4_ + SMF hydrogels. (**K**) Porosity of the three hydrogels. (**L**) Swelling ratios of the three hydrogels. (**M**, **N**) Compressive strength and compressive modulus of the three hydrogels, respectively. The statistical analysis revealed significant differences (*P < 0.05, **P < 0.01, ***P < 0.001, ****P < 0.0001).

The GelMA–Fe_3_O_4_ hydrogel system was synthesized by uniformly dispersing MAA–Fe_3_O_4_ nanoparticles (400 μg/mL) within the GelMA matrix and crosslinking under visible light (405 nm, 60 seconds) using LAP. As control groups, pure GelMA and physically mixed GelMA/Fe_3_O_4_ hydrogels were prepared simultaneously (Figure 3A). The resulting hydrogels exhibited a 3D structure; the GelMA hydrogel was transparent and white, whereas the GelMA–Fe_3_O_4_ hydrogel appeared light brown due to the incorporation of MAA–Fe_3_O_4_ particles (Figure 3B). The EDS mapping revealed the distribution of various elements within the hydrogels (Figure 3C and D). Iron elements were uniformly dispersed on the surface of the functionalized GelMA–Fe_3_O_4_ hydrogel, whereas significant agglomeration of iron elements was observed in the physically mixed GelMA/Fe_3_O_4_ hydrogel.

The SEM results, following freeze-drying, indicated that the incorporation of nanoparticles resulted in roughened pore walls of the hydrogel and a slight reduction in pore size (Figure 3E). During the *in vitro* degradation process, spanning from 0 to 60 days, the hydrogel transitioned from an intact porous network to surface erosion, enhanced pore interconnectivity, and ultimately to network disintegration and fragmentation (Figure 3F). Quantitative analysis of the SEM images revealed that the porosity of GelMA–Fe_3_O_4_ was greater than that of GelMA/Fe_3_O_4_ (Figure 3K). The XRD analysis demonstrated that the characteristic diffraction peaks of Fe_3_O_4_ were preserved in both GelMA/Fe_3_O_4_ and GelMA–Fe_3_O_4_, with only minor shifts in peaks observed when compared to pure Fe_3_O_4_ nanoparticles, indicating stable crystal phases alongside interfacial stress (Figure 3G). The FTIR results illustrated that the absorption peaks of the three sample groups were highly consistent, confirming that the incorporation and integration of magnetic nanoparticles did not significantly alter the types of chemical bonds and molecular conformations within the polymer network (Figure 3H). Furthermore, the VSM test indicated that although the saturation magnetization of the GelMA–Fe_3_O_4_ hydrogel was slightly lower than that of the physically mixed GelMA/Fe_3_O_4_ hydrogel, both types exhibited excellent superparamagnetic properties (Figure 3I).

The degradation rate measurement of the composite hydrogels (Figure 3J) revealed that the mass loss of each hydrogel was approximately 50–60% after 40 days. Notably, the GelMA–Fe_3_O_4_ hydrogel exhibited the slowest mass loss, while the SMF had no significant effect on hydrogel degradation. The hydrogel swelling experiment (Figure 3L) demonstrated that the incorporation of nanoparticles reduced the water absorption rate, with the GelMA–Fe_3_O_4_ hydrogel showing the lowest absorption rate. Additionally, mechanical property testing of the hydrogel (Figure 3M and 3N) confirmed that the covalent grafting of MAA–Fe_3_O_4_ significantly enhanced both the compressive modulus and compressive strength of the hydrogel.

### Biocompatibility and cell proliferation

**Figure 4:**
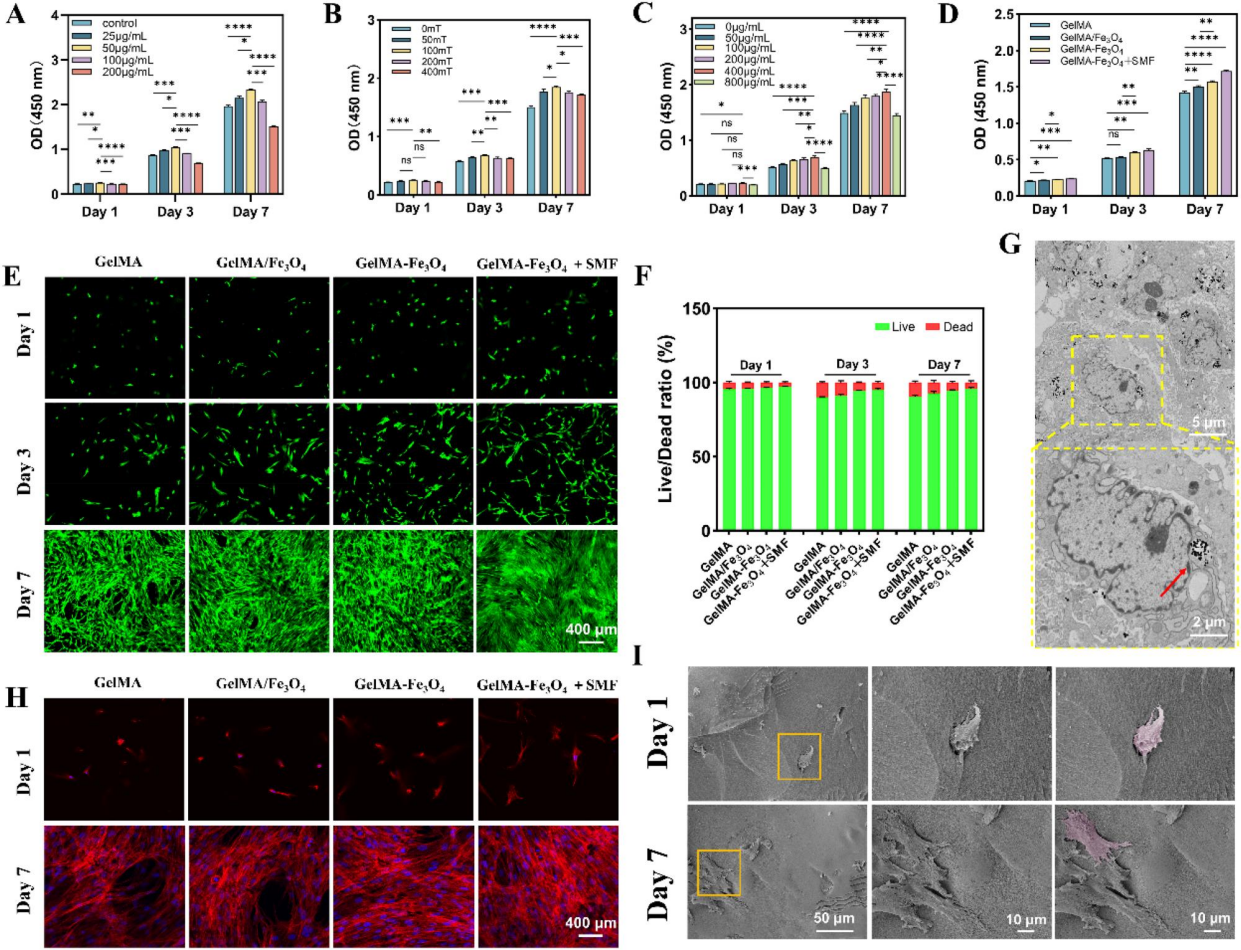
Biocompatibility assessment of composite hydrogels. (**A**) CCK-8 results of hDFSCs co-cultured with different concentrations of Fe_3_O_4_ for 1, 3 and 7 days. (**B**) CCK-8 results of hDFSCs co-cultured with 50 μg/mL Fe_3_O_4_ under different magnetic field intensities for 1, 3 and 7 days. (**C**) CCK-8 assay of hDFSCs co-cultured with GelMA–Fe_3_O_4_ hydrogels with different Fe_3_O_4_ loadings for 1, 3 and 7 days. (**D**) CCK-8 results of hDFSCs co-cultured with various types of hydrogels for 1, 3 and 7 days. (**E**) Live/dead staining images of hDFSCs co-cultured with various types of hydrogels for 1, 3 and 7 days (scale bar = 400 μm). (**F**) Quantitative analysis of live/dead staining images. (**G**) TEM images of hDFSCs after phagocytosis of Fe_3_O_4_ nanoparticles (top 5 μm, bottom 2 μm). (**H**) Fluorescence images of F-actin (red) of hDFSCs cultured on various hydrogel groups on days 1 and 7 (scale bar 400 μm). (**I**) SEM images of hDFSCs cultured in GelMA–Fe_3_O_4_ hydrogel on days 1 and 7 (pink-marked areas indicate cell spreading regions; left 50 μm, middle 10 μm, right 10 μm). (*P < 0.05, **P < 0.01, ***P < 0.001, ****P < 0.0001).

Good biocompatibility has consistently been a fundamental requirement in the design of novel bone tissue engineering materials [23]. In this experiment, the hDFSCs used were extracted and identified (Supplementary Figure S1), confirming their stem cell characteristics. To evaluate the optimal stimulation conditions, the CCK-8 kit was employed to assess the effects of MAA–Fe_3_O_4_ nanoparticle concentration (0–100 μg/mL), SMF intensity (0–400 mT), and particle loading in the composite hydrogel (0–800 μg/mL) on the proliferation of hDFSCs. The results indicated that MAA–Fe_3_O_4_ nanoparticles at a concentration of 50 μg/mL, subjected to a magnetic field of 100 mT, and incorporated into a GelMA–Fe_3_O_4_ hydrogel at a loading concentration of 400 μg/mL exhibited the highest optical density (OD) values on both day 3 and day 7 (Figure 4A–C). Therefore, these conditions were selected for subsequent studies.

The composite hydrogel exhibited a significant increase in OD values over time, as indicated by the CCK-8 assay. Notably, the GelMA–Fe_3_O_4_ + SMF group demonstrated the highest proliferation rate (Figure 4D). Live/dead staining and the CLSM observations revealed that the survival-to-death cell ratios were comparable across all groups on day 1. However, on days 3 and 7, the GelMA–Fe_3_O_4_ group displayed significantly higher cell viability compared to the physically mixed GelMA/Fe_3_O_4_ group. Importantly, the application of SMF did not lead to an increase in dead cells; rather, it further enhanced cell proliferation (Figure 4E and F), with the GelMA–Fe_3_O_4_ + SMF group exhibiting the best biocompatibility.

### Observation of cell morphology and nanoparticle uptake

The TEM observations (Figure 4G) demonstrated that hDFSCs could phagocytose MAA–Fe_3_O_4_ nanoparticles through endocytosis within 1 day of culture, with these particles distributed throughout the cytoplasm without altering the morphology of organelles. F-actin staining combined with CLSM observations (Figure 4H) further indicated that cells in the control group (pure GelMA) maintained a spherical morphology. In contrast, cells in the GelMA–Fe_3_O_4_ and GelMA–Fe_3_O_4_ + SMF groups exhibited significant stress fibers and an elongated morphology from the first day. By the seventh day, the hDFSCs influenced by the SMF had formed a denser and more robust network of fibers, indicating enhanced inter-pseudopodial connections. SEM observations (Figure 4I) revealed that the hDFSCs in the GelMA–Fe_3_O_4_ hydrogel began to extend initial pseudopods on the first day, and by the seventh day, there was a significant increase in both the number and length of the pseudopods, with the cells widely spread out.

### SMF-enhanced *in vitro* osteogenic differentiation

**Figure 5:**
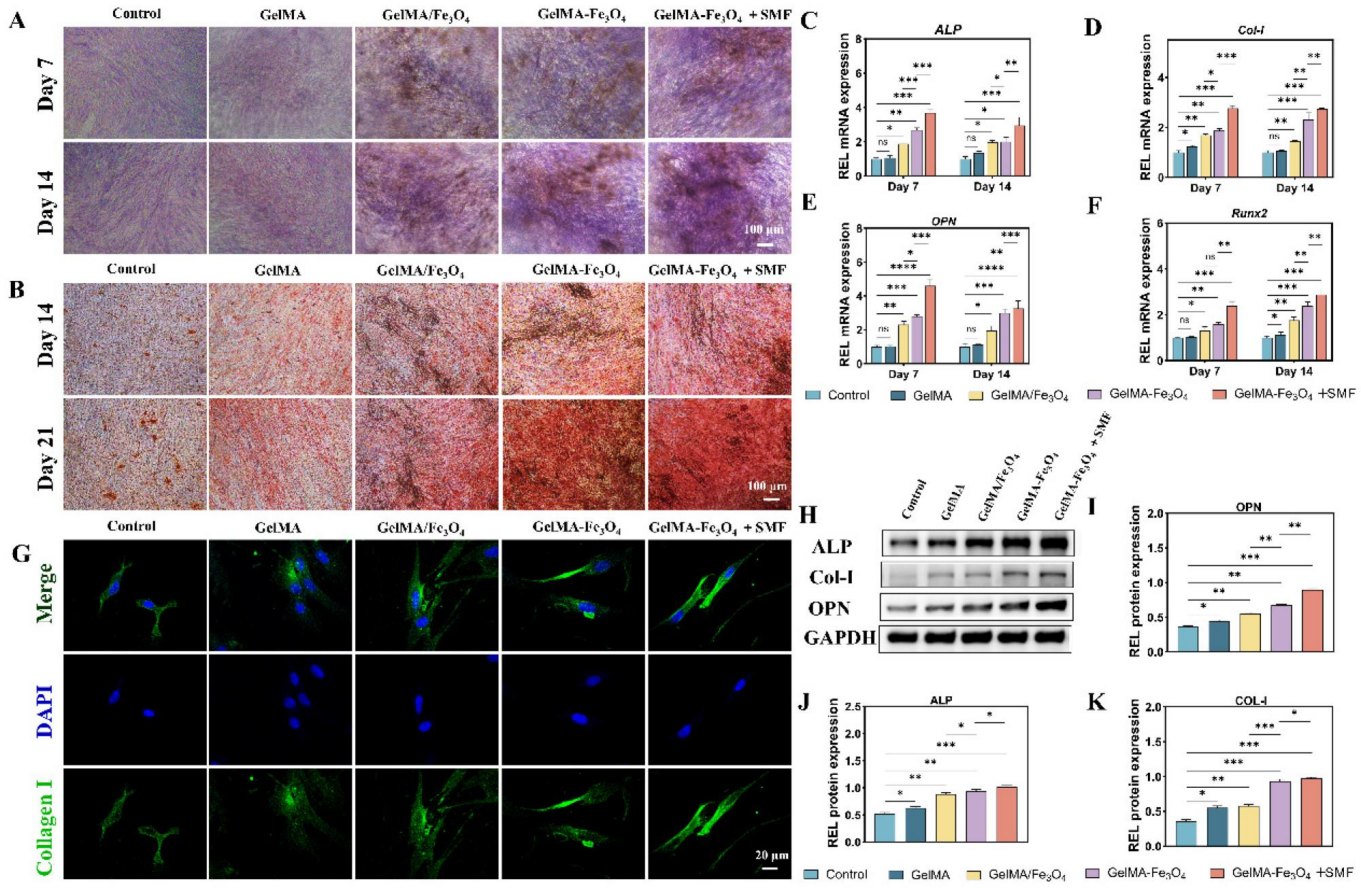
Osteogenic characteristics of hDFSCs cultured *in vitro* with various types of hydrogels. (**A**) ALP staining images on day 7 and day 14 (scale bar = 100 μm). (**B**) ARS staining images on day 14 and day 21 (scale bar = 100 μm). (**C–F**) Expression of osteogenesis-related genes ALP, COL-I, OPN, and RUNX2 on day 7 and day 14. (**G**) On day 14, COL-I immunofluorescence images (green, scale bar = 20μm). (**H**) Expression of osteogenesis-related proteins ALP, COL-I and OPN on day 14. (**I–K**) Quantitative analysis of osteogenesis-related proteins ALP, COL-I and OPN on day 14 (*P < 0.05, **P < 0.01, ***P < 0.001, ****P < 0.0001).

The biomineralization process mediated by osteoblasts is a critical step in the repair of bone tissue defects [24]. Qualitative and quantitative analysis through ALP staining (Figure 5A, Supplementary Figure S2) demonstrated that the functionalized GelMA–Fe_3_O_4_ hydrogel exhibited significantly stronger ALP activity compared to the physically mixed GelMA/Fe_3_O_4_ group at both 7 and 14 days. Moreover, the intensity of ALP staining in the GelMA–Fe_3_O_4_ group was further enhanced under the influence of a 100 mT SMF. Qualitative ARS staining (Figure 5B) indicated that the number and area of mineralized nodules formed in the GelMA–Fe_3_O_4_ group stimulated by SMF were significantly greater than those in the non-magnetic field group.

The quantitative real-time polymerase chain reaction (RT-qPCR) results (Figure 5C–F) demonstrated that at both 7 and 14 days, the mRNA expression levels of *ALP, Runx2, COL-I,* and *OPN* in the GelMA–Fe_3_O_4_ + SMF group were significantly upregulated compared to the GelMA/Fe_3_O_4_ and GelMA–Fe_3_O_4_ groups. Furthermore, COL-I immunofluorescence staining (Figure 5G, Figure S1) confirmed that SMF enhanced the secretion of osteogenic proteins. Additionally, Western blot and semi-quantitative analysis (Figure 5H–K) revealed that the protein levels of ALP, COL-I, and OPN were highest in the GelMA–Fe_3_O_4_ + SMF group, significantly exceeding those in the other groups.

### SMF promotes *in vivo* bone regeneration of hDFSCs encapsulated in GelMA–Fe_3_O_4_ hydrogel

**Figure 6:**
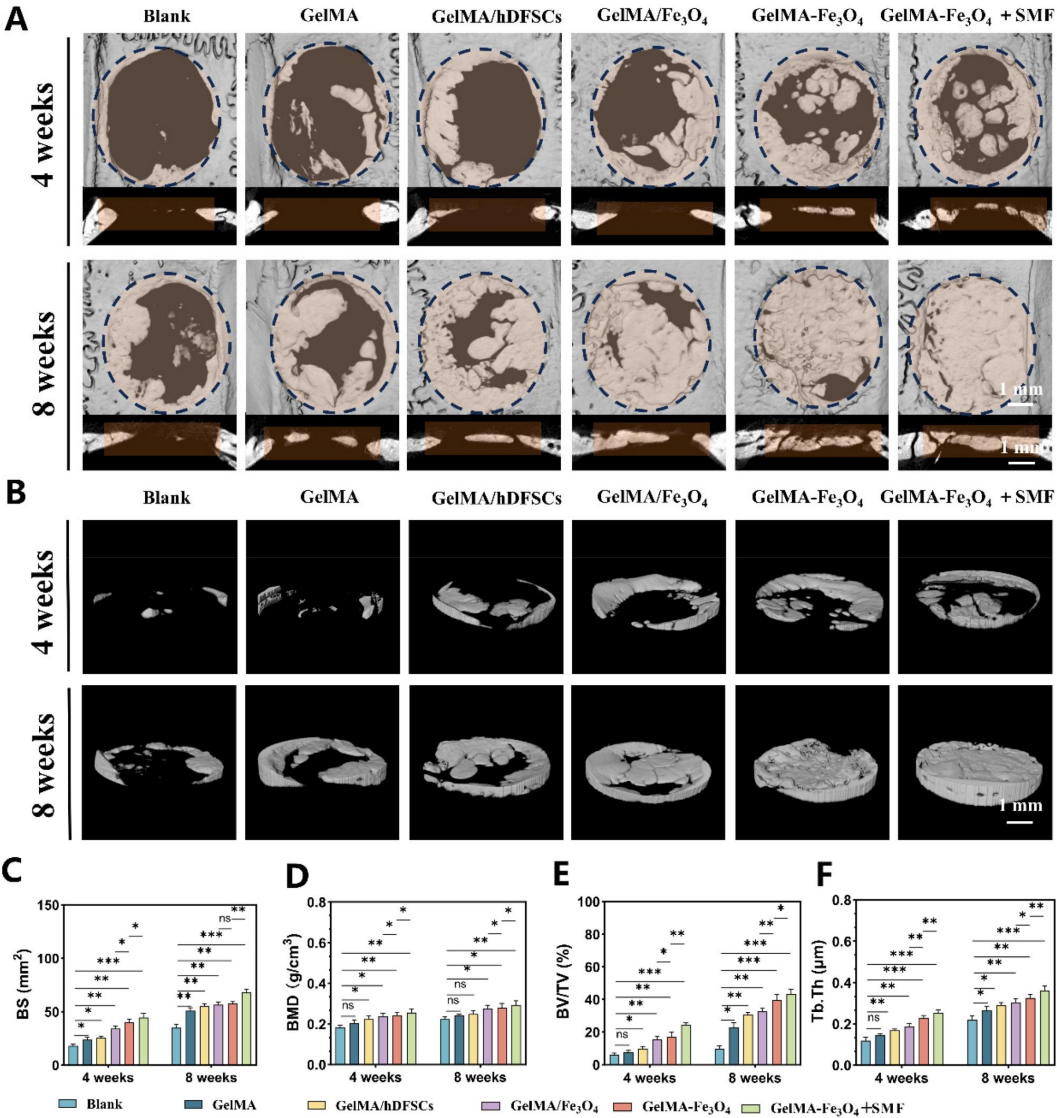
*In vivo* bone regeneration evaluation of different hydrogels in a rat calvarial defect model. (**A**) Micro-CT images at 4 and 8 weeks post-operation (scale bar = 1mm). (**B**) 3D reconstructed CT images at 4 and 8 weeks post-operation (scale bar = 1mm). (**C–F**) Quantitative analysis of BS, BMD, BV/TV, and Tb.Th. at 4 and 8 weeks post-operation. (*P < 0.05, **P < 0.01, ***P < 0.001, ****P < 0.0001).

Based on the *in vitro* experimental results, we compared the osteogenic effects of various groups, including the blank control, GelMA, GelMA + hDFSCs, GelMA/Fe_3_O_4_ + hDFSCs, GelMA–Fe_3_O_4_ + hDFSCs, and GelMA–Fe_3_O_4_ + hDFSCs + SMF, using a 5 mm SD rats calvarial defect model. Micro-CT 3D reconstruction (Figure 6A and B) indicated that at the 4th week, only minimal new bone formation was observed at the defect margins in both the blank control and GelMA groups. In contrast, the GelMA–Fe_3_O_4_ group exhibited multiple discrete bone centers in the central region, while the GelMA–Fe_3_O_4_ + SMF group demonstrated the presence of multi-site bone centers characterized by a more continuous and thicker structure. Quantitative analysis using Micro-CT (Figure 6C–E) revealed that in the SMF group, the BV/TV reached 24 ± 3% at 4 weeks (4.7 times that of the control group) and increased to 41 ± 5% at 8 weeks, with the Tb.Th results significantly higher than those of the other groups (Figure 6F).

**Figure 7:**
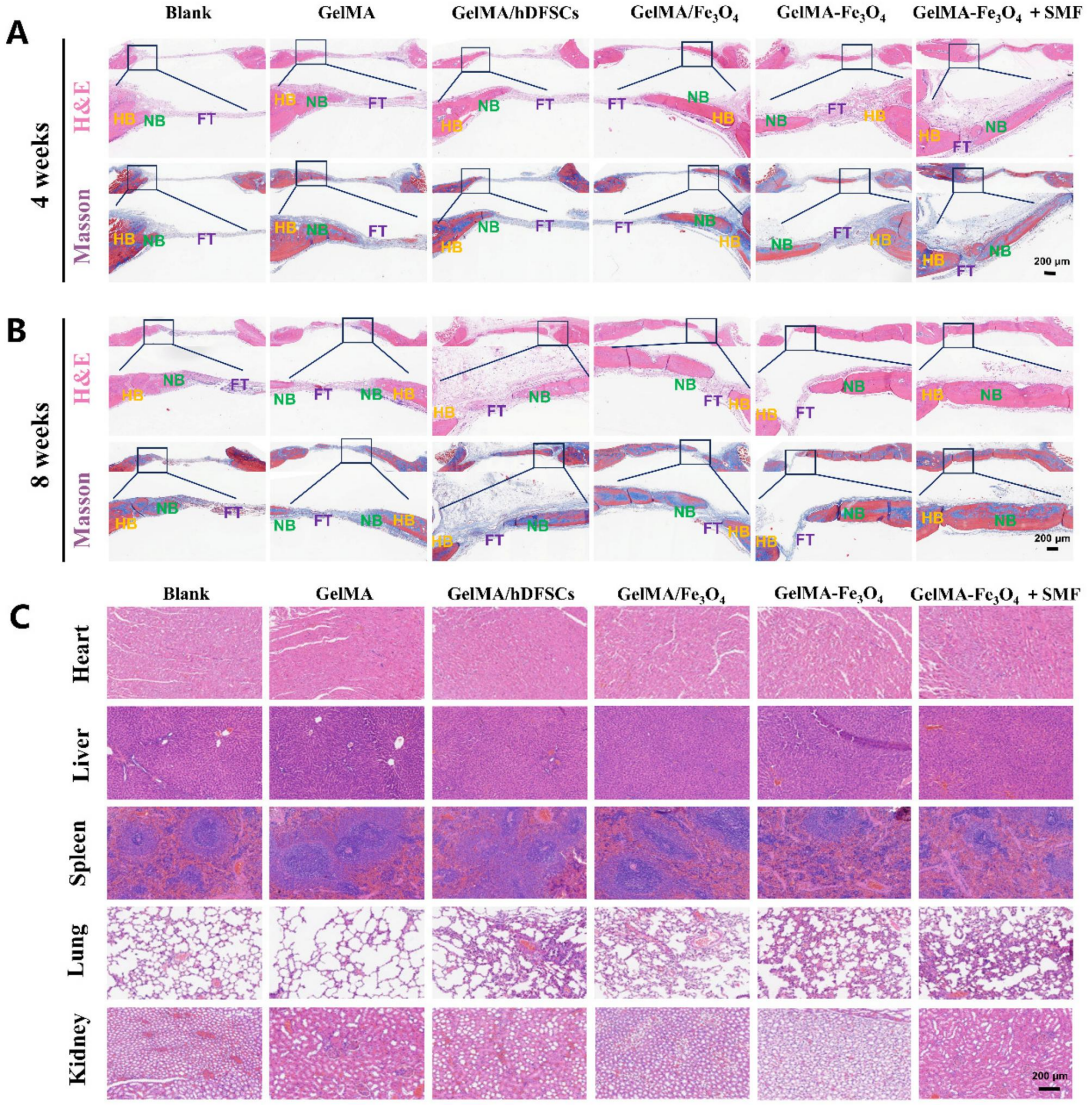
*In vivo* bone regeneration and biosafety evaluation in histological analysis. (**A**) H&E and Masson staining of the defect area at 4 weeks post-operation (HB, host bone; NB, new bone; FT, fibrous tissue; scale bar 200 μm). (**B**) H&E and Masson staining of the defect area at 8 weeks post-operation (scale bar 200 μm). (**C**) H&E staining of the heart, liver, spleen, lung and kidney of rats 8 weeks after hydrogel implantation (scale bar 200 μm) (*P < 0.05, **P < 0.01, ***P < 0.001, ****P < 0.0001).

**Figure 8:**
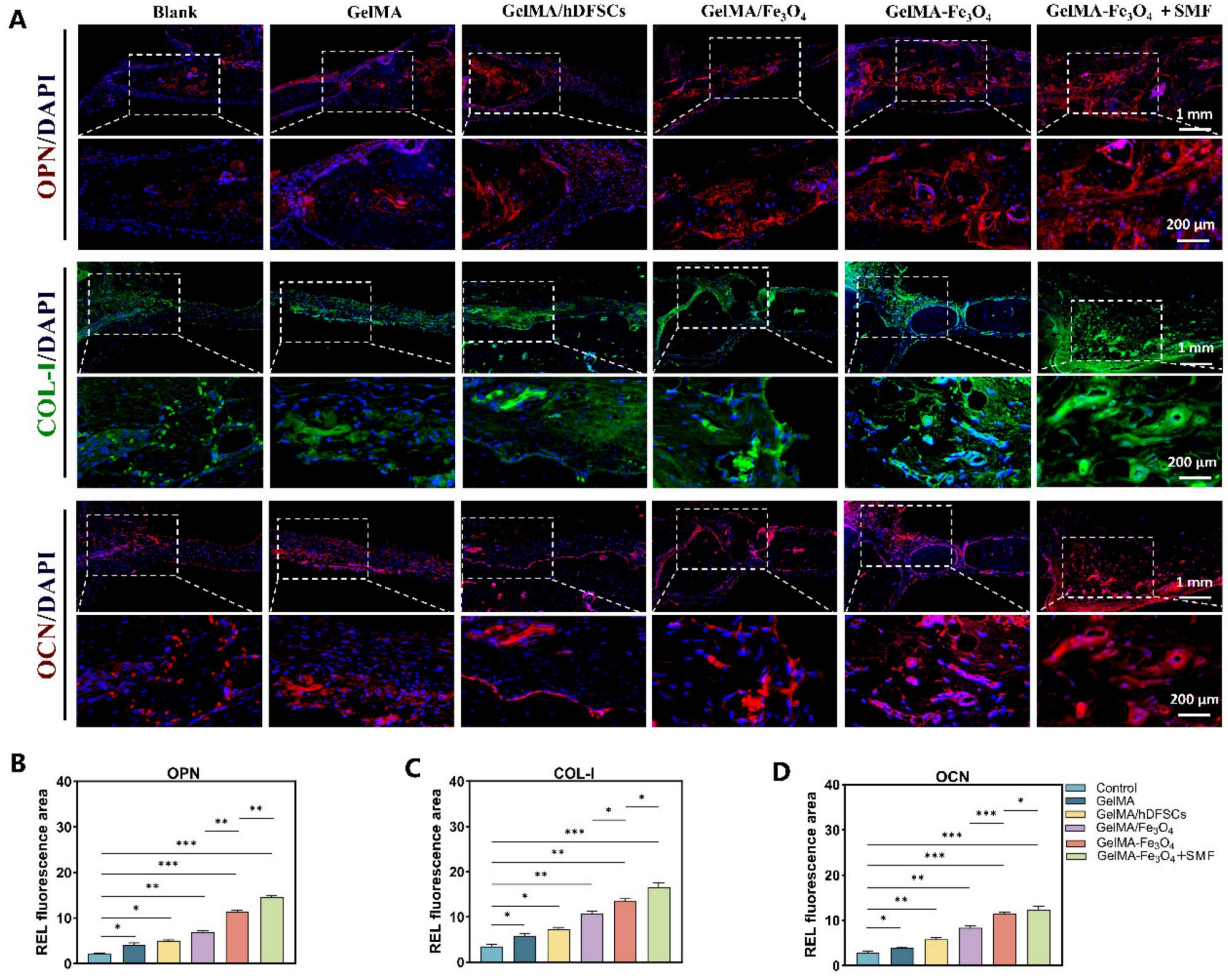
Histological immunofluorescence evaluation of osteogenesis-related proteins. (**A**) Representative images of OPN (red), COL-I (green), OCN (red) and nuclei (DAPI, blue) at 8 weeks post-operation (upper row scale bar 1 mm; lower row scale bar 200 μm). (**B–D**) Quantitative analysis of the positive signal areas of OPN, COL-I and OCN (*P < 0.05, **P < 0.01, ***P < 0.001, ****P < 0.0001).

H&E/Masson staining (Figure 7A and B) further confirmed that at 4 weeks, multiple discrete bone centers were observed in both the GelMA–Fe_3_O_4_ and GelMA–Fe_3_O_4_ + SMF groups. After 8 weeks, only the SMF group exhibited complete filling of the defect area with dense new bone bridging, while the other groups demonstrated limited marginal repair or incomplete filling. H&E staining of major organs at 4 and 8 weeks post-operation indicated no inflammatory infiltration or pathological damage (Supplementary Figure S4, 4 weeks; Figure 7C, 8 weeks). Immunofluorescence for COL-I, OPN and OCN at 8 weeks (Figure 8A–D) revealed that the positive areas of the three osteogenic markers in the GelMA–Fe_3_O_4_ + SMF group were significantly higher than those in the non-magnetic field and control groups. Additionally, the positive areas of osteogenic markers in the GelMA–Fe_3_O_4_ group were superior to those in the GelMA/Fe_3_O_4_ group.

## Discussion

Although the traditional strategy of combining stem cells with biomaterials can promote bone regeneration, it often depends on extensive *in vitro* cell expansion and differentiation, which is time-consuming and may induce immune rejection [25, 26]. The hDFSCs, characterized by their high proliferation rate, excellent osteogenic potential, low immunogenicity, and the convenience of being obtainable from a single wisdom tooth, have emerged as ideal seed cells for maxillofacial bone repair [27–29]. However, the development of rationally designed biomaterial carriers that can achieve durable and stable *in situ* osteogenesis while minimizing cell usage remains a challenge. While Fe_3_O_4_ nanoparticles have garnered significant attention due to their unique superparamagnetic properties and osteogenic activity [30], traditional non-functionalized Fe_3_O_4_ still faces challenges including poor biocompatibility, agglomeration, insufficient interaction with the matrix [31], and potential toxicity to surrounding tissues due to “burst release” phenomena at high concentrations [32, 33]. These key limitations restrict their application efficacy. To address these challenges, in this study, we innovatively constructed covalently crosslinked GelMA–Fe_3_O_4_ composite hydrogels using the MAA functionalization strategy. Compared to the physically mixed GelMA/Fe_3_O_4_ control group, the functionalized composite hydrogel showed more uniform distribution of Fe_3_O_4_ in EDS analysis, effectively preventing agglomeration.

Long-term safety assessment of biomedical materials is crucial for clinical translation [34]. Low-dose Fe_3_O_4_ nanoparticles have been FDA-approved for clinical use, with their *in vivo* metabolism mechanism well-elucidated: the nanoparticles are primarily accumulated in the liver and spleen through the mononuclear-macrophage system, subsequently degraded into iron ions in the acidic environment of lysosomes (pH 4.5–5.0) [35], and then integrated into normal iron metabolism cycles via transferrin and ferritin pathways, effectively avoiding oxidative stress induced by free iron ions [36]. Their complete metabolic cycle typically requires several weeks to months [35, 37], and systemic toxicity may only occur when iron intake exceeds 20–30 mg Fe/kg body weight [38], while the local application dose in our study is far below this safety threshold. We verified the biosafety of the GelMA–Fe_3_O_4_ composite hydrogel through multidimensional experiments: *in vitro* experiments confirmed no significant cytotoxicity at 50 μg/mL concentration; TEM observation showed that hDFSCs could effectively internalize and metabolize Fe_3_O_4_ particles; histological analysis of major organs at 4 and 8 weeks *in vivo* detected no significant pathological changes. Furthermore, the MAA functionalization strategy significantly enhanced the dispersion stability and controlled degradation of Fe_3_O_4_ nanoparticles, further reducing their potential biological risks.

Cell proliferation and adhesion are core indicators for evaluating the biocompatibility of biomaterials [39]. The microstructure of the material, particularly pore size, directly influences cell survival and functional expression [40]. Due to its biomimetic 3D network structure and excellent biocompatibility, GelMA hydrogel has been widely utilized in the 3D culture of stem cells and tissue engineering [41]. However, the high crosslinking density and small pore size of traditional GelMA hydrogels limit nutrient exchange and cell migration, adversely affecting the survival rate and proliferation capacity of encapsulated cells [42, 43]. In the results of this study, live/dead staining and CCK-8 assays confirmed that hDFSCs encapsulated in GelMA–Fe_3_O_4_ hydrogels exhibited excellent adhesion properties, higher cell density and larger cell spreading areas. The SEM and CLSM observations further revealed that cells formed more pseudopodia and complex cytoskeletal networks within the magnetic hydrogels. Overall, these findings indicate that the composite structure of GelMA–Fe_3_O_4_ hydrogels better supports cell growth, proliferation and adhesion, thereby providing a favorable environment for cell development and proliferation.

Bone regeneration involves the precise regulation of various osteogenic genes and proteins [44]. ALP, OPN, Runx2 and COL-I are recognized as key transcription factors and markers of osteogenic differentiation [45–48]. The RT-qPCR results of this study demonstrated that GelMA–Fe_3_O_4_ hydrogel significantly upregulated the expression of osteogenesis-related genes in hDFSCs, with this upregulation further enhanced by the application of an SMF. Western blot analysis confirmed that the changes at the protein level were consistent with the trends observed in gene expression. This biological phenomenon can be explained through the multi-level mechanisms of SMF action: at the molecular level, SMF may activate mechanosensitive ion channels such as Piezo1, leading to increased intracellular Ca^2+^ concentration and subsequent activation of downstream osteogenic signaling pathways [49, 50]; at the cellular level, the magneto-mechanical stimulation produced by the interaction between SMF and Fe_3_O_4_ nanoparticles optimizes cytoskeletal organization and cell adhesion structures [51], which is consistent with our observation of hDFSCs forming more complex pseudopodial networks and increased COL-I secretion. These *in vitro* findings provide important evidence for understanding the molecular basis of how SMFs and covalently crosslinked magnetic hydrogels synergistically promote bone regeneration.

*In vivo* experiments revealed a unique osteogenic phenomenon: the newly formed bone exhibited a multi-site, discrete “bone center” morphology, distinctly differing from the traditional bone repair pattern that gradually converges from the defect edges toward the center [52]. Experimental results showed that at 4 weeks, the GelMA–Fe_3_O_4_ + SMF treatment group exhibited significantly enhanced bone formation with discontinuous distribution characteristics, while the control groups only produced limited new bone tissue in restricted areas around the defect periphery. This characteristic of multi-site synchronous bone regeneration may be attributed to three synergistic mechanisms: firstly, the slow and sustained release of MAA–Fe_3_O_4_ nanoparticles within the hydrogel creates multiple osteogenic microenvironments [53, 54]; secondly, the synergistic effect between the SMF and Fe_3_O_4_ nanoparticles generates local magnetic field gradients, promoting *in situ* proliferation of encapsulated cells and enhancing their matrix adhesion, facilitating their aggregation and differentiation into osteoblasts at multiple sites [10, 55]; thirdly, the paracrine effect of hDFSCs, through the secretion of exosomes and osteogenic factors, may recruit host cells to participate in the osteogenic process, thereby amplifying the repair effect [56, 57]. Compared with traditional hydrogels, the GelMA–Fe_3_O_4_ hydrogel loaded with hDFSCs demonstrated the ability to simultaneously regenerate bone at multiple sites, with nearly complete repair of bone defects observed at 8 weeks. This strategy significantly improves the efficiency and quality of bone regeneration through multi-center synchronous osteogenesis.

Although this study confirms the potential of functionalized magnetic hydrogel systems in bone tissue engineering, some limitations still exist. First, the molecular mechanisms by which MAA–Fe_3_O_4_ nanoparticles and SMF synergistically enhance hDFSCs osteogenic differentiation need further investigation, particularly how they regulate osteogenesis-related signaling pathways remains poorly understood; second, the relative contributions of transplanted hDFSCs versus endogenous host cells in the formation of multi-site bone centers remain unclear. These issues will be the focus of our subsequent research to further optimize the application potential of this magnetic hydrogel system. In summary, this functionalized magnetic hydrogel system is expected to overcome the limitations of traditional osteogenic strategies, offering an innovative solution for the rapid repair of large-scale bone defects, with promising prospects for clinical translation.

## Conclusion

This study presents the development of an injectable, photo-crosslinked GelMA magnetic hydrogel system that is covalently crosslinked with MAA-functionalized Fe_3_O_4_ nanoparticles. By encapsulating hDFSCs derived from discarded dental follicles and utilizing the synergistic effect of a low-intensity SMF (100 mT), the system effectively repaired cranial bone defects in SD rats. This innovative system significantly enhanced the proliferation, adhesion and osteogenic differentiation of hDFSCs by creating an optimized 3D microenvironment, which facilitated the formation of a unique multi-site ‘bone centers’ osteogenic pattern *in vivo*. This approach overcomes the traditional limitations of unidirectional osteogenesis, characterized by an ‘edge-to-center’ paradigm. The results indicated that the system successfully achieved complete bone bridge reconstruction, as evidenced by both radiological and histological analyses, without the need for exogenous growth factors. This offers a novel therapeutic strategy for addressing large-volume craniomaxillofacial bone defects that is efficient, controllable, and promising in alleviating the shortage of clinical seed cells.

## Supplementary Material

rbag023_Supplementary_Data
