# Supplementary material for: Functionalized magnetic hydrogel encapsulation of human dental follicle stem cells under a static magnetic field enhances multi-site bone regeneration
Source: Regen Biomater. 2026 Mar 7;13:rbag023. doi: 10.1093/rb/rbag023 (PMC13037764; doi:10.1093/rb/rbag023)
Supplement: rbag023_Supplementary_Data [file rbag023_supplementary_data.docx]

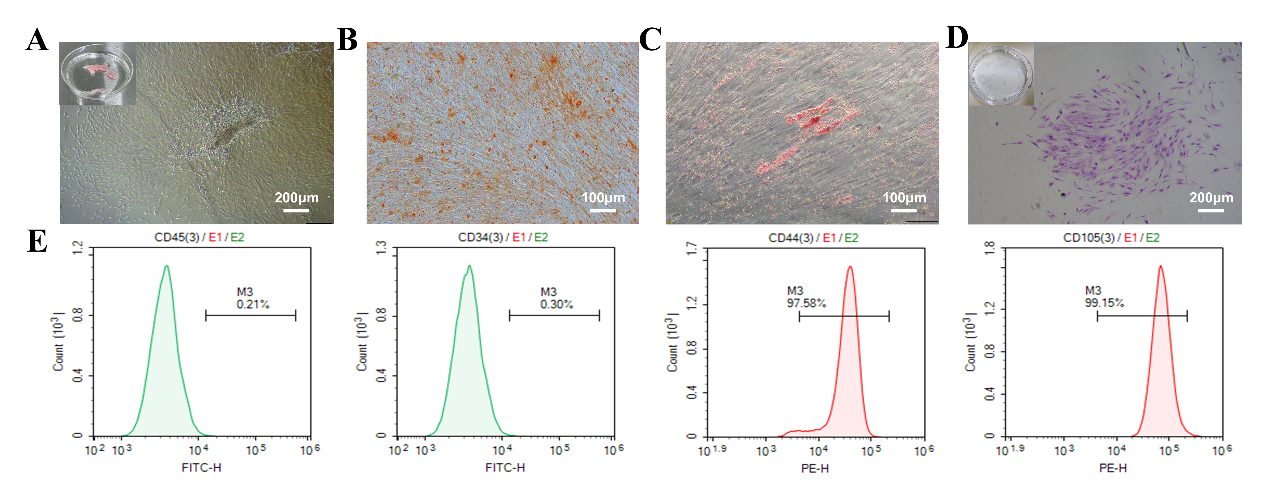


**Figure S1:** Characteristics and differentiation potential of hDFSCs. (A) Primary cultured hDFSCs (scale bar = 200 μm). (B) Alizarin Red S (ARS) staining after 21 days of osteogenic induction (scale bar = 100 μm). (C) Oil Red O staining after 28 days of adipogenic induction (scale bar = 100 μm). (D) Colony formation assay (scale bar = 200 μm). (E) Flow cytometry analysis of surface markers (CD45, CD34, CD44, and CD105) of hDFSCs.


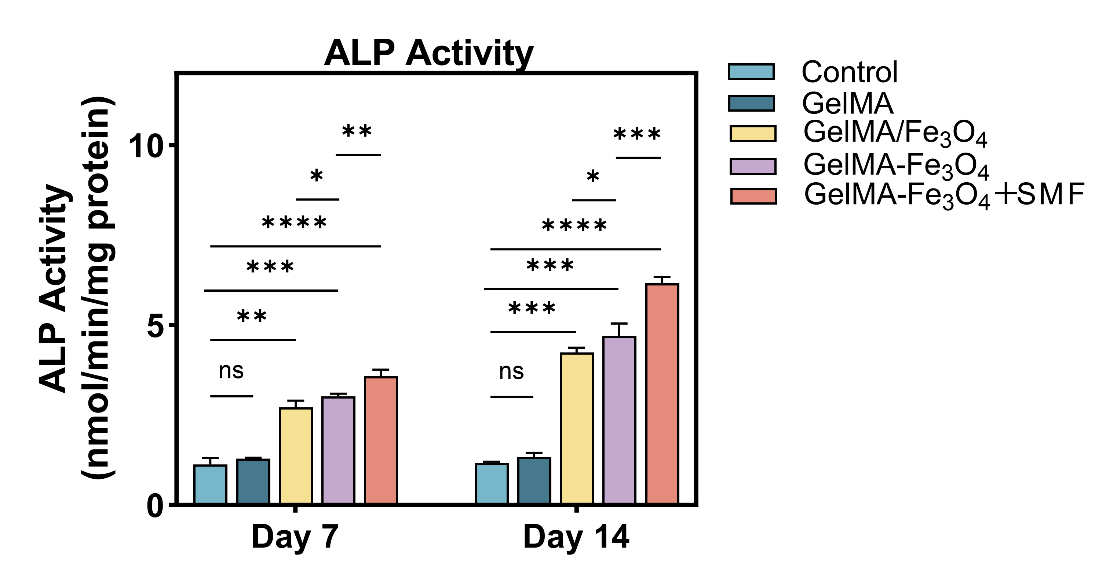


**Figure S2:** Evaluation of osteogenic function: ALP activity on day 7 and day 14.
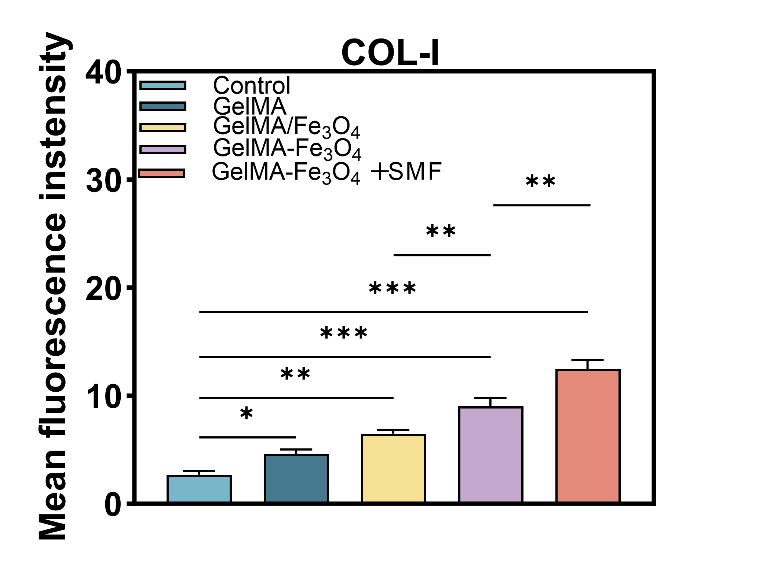


**Figure S3:** Quantitative fluorescence analysis of COL-I.


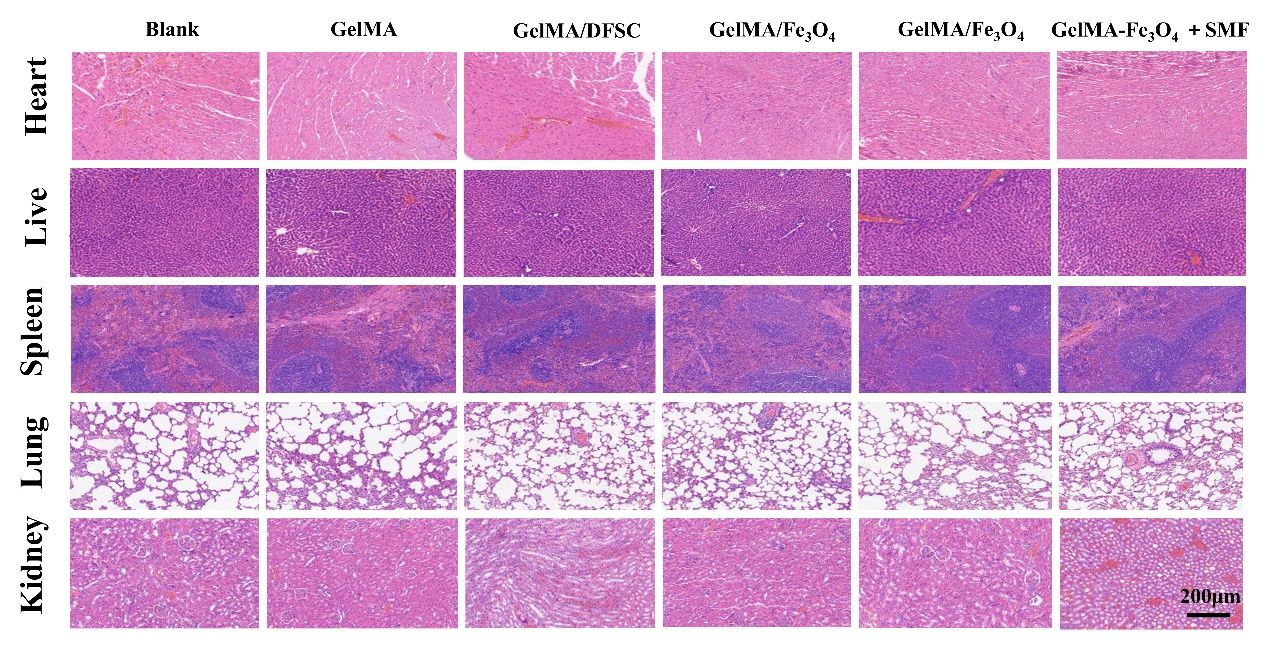


**Figure S4:** H&E staining of heart, liver, spleen, lung, and kidney in rats 4 weeks after hydrogel implantation (scale bar 200μm).
